# Airborne Detection and Quantification of Swine Influenza A Virus in Air Samples Collected Inside, Outside and Downwind from Swine Barns

**DOI:** 10.1371/journal.pone.0071444

**Published:** 2013-08-08

**Authors:** Cesar A. Corzo, Marie Culhane, Scott Dee, Robert B. Morrison, Montserrat Torremorell

**Affiliations:** 1 Department of Veterinary Population Medicine, College of Veterinary Medicine, University of Minnesota, Saint Paul, Minnesota, United States of America; 2 University of Minnesota Veterinary Diagnostic Laboratory, Saint Paul, Minnesota, United States of America; 3 Pipestone Veterinary Clinic, Pipestone, Minnesota, United States of America; Wageningen University and Research Centre, Netherlands

## Abstract

Airborne transmission of influenza A virus (IAV) in swine is speculated to be an important route of virus dissemination, but data are scarce. This study attempted to detect and quantify airborne IAV by virus isolation and RRT-PCR in air samples collected under field conditions. This was accomplished by collecting air samples from four acutely infected pig farms and locating air samplers inside the barns, at the external exhaust fans and downwind from the farms at distances up to 2.1 km. IAV was detected in air samples collected in 3 out of 4 farms included in the study. Isolation of IAV was possible from air samples collected inside the barn at two of the farms and in one farm from the exhausted air. Between 13% and 100% of samples collected inside the barns tested RRT-PCR positive with an average viral load of 3.20E+05 IAV RNA copies/m^3^ of air. Percentage of exhaust positive air samples also ranged between 13% and 100% with an average viral load of 1.79E+04 RNA copies/m^3^ of air. Influenza virus RNA was detected in air samples collected between 1.5 and 2.1 Km away from the farms with viral levels significantly lower at 4.65E+03 RNA copies/m^3^. H1N1, H1N2 and H3N2 subtypes were detected in the air samples and the hemagglutinin gene sequences identified in the swine samples matched those in aerosols providing evidence that the viruses detected in the aerosols originated from the pigs in the farms under study. Overall our results indicate that pigs can be a source of IAV infectious aerosols and that these aerosols can be exhausted from pig barns and be transported downwind. The results from this study provide evidence of the risk of aerosol transmission in pigs under field conditions.

## Introduction

Influenza A virus (IAV) is a negative sense single stranded RNA virus belonging to the *Orthomyxovirideae* family [1]. In swine, IAV causes respiratory disease characterized by anorexia, fever, sneezing, coughing, rhinorrhea and lethargy and the febrile state in pregnant animals can lead to abortions [2], [3]. The disease is characterized by low mortality but high morbidity and decreased growth performance which results in increased pig weight variation. Besides the effects on animal health, IAV is an important zoonotic pathogen and pigs can be a reservoir and a source of novel reassortants [4], including viruses of pandemic potential. Therefore IAV has implications for both animal and public health, and understanding transmission of IAV in animal populations is crucial to prevent zoonotic infections.

Infected pigs can shed virus through nasal secretions for approximately 5 to 7 days allowing transmission to occur by direct nose-to-nose contact. Commonly, sudden respiratory disease outbreaks follow the introduction of infected pigs originating from infected sources [5] resulting in the introduction of new viruses. However, reports of respiratory illness may not always be related to pig introduction. The airborne route can also play a role in viral spread [3], [6]. Risk factor studies in Canada [7] and Belgium [8] found that the likelihood of a pig farm being positive for influenza was significantly associated with pig farm density, suggesting that other routes, such as airborne, can play a role in between herd transmission. Recently, pig farm proximity to turkey flocks has been associated with turkey seropositivity to swine-origin IAV which suggest that the airborne route may have played a role in transmission [9]. Aerosol transmission of IAV has also been reported in humans, [10]–[12] mice, guinea pigs, ferrets and chickens [13]–[17] indicating that airborne transmission of IAV plays an important role in the ecology of influenza. However, the importance of the airborne transmission route in pigs remains under debate despite the fact that airborne dissemination in pigs has been documented for foot and mouth disease virus, pseudorabies virus, *Mycoplasma hyopneumoniae* and porcine reproductive and respiratory virus (PRRSV) [18]–[24].

Recently, IAV was detected in aerosols generated from infected pigs vaccinated for IAV and [25] and also in pigs with passive immunity [26]. Corzo et al. [27] reported an association between detecting virus in nasal secretions and the likelihood of detecting virus in the air. In these studies, virus could be readily detected in air samples collected during the acute infection phase suggesting that acutely infected pigs can be a substantial source of infectious virus. However, the implications of these studies for field settings can only be speculated. To the authors’ knowledge there is no data on the detection of IAV in aerosols generated by pigs under field conditions. Thus, the objectives of this study were to determine whether IAV could be detected in air samples collected in swine farms and downwind from them, and provide an estimate for quantities of viral load found in swine aerosols under field conditions.

## Materials and Methods

### Ethics Statement

Procedures and protocols used in this study were approved by the University of Minnesota Institutional Animal Care and Use Committee (IACUC) and the Institutional Biosafety Committee (IBC).

### Farm Identification and Selection

Four pig farms were selected for this study (farms 1 through 4) during the months of April, September and October 2011 by contacting veterinarians that care for pigs in Southern Minnesota and Northern Iowa. Veterinarians were asked to alert the investigators upon sudden onset of respiratory clinical signs in growing pig populations suggestive of acute influenza like illness (i.e. rapid onset of widespread dry hacking cough, sneezing, rhinorrhea, anorexia and lethargy). Farms were included in the study if the veterinarian had a presumptive diagnosis suggestive of influenza or was able to collect samples and confirm the presumptive diagnosis within 2 to 4 days from the onset of clinical signs, and was able to communicate with the investigators within 2 to 3 days from the onset of disease.

Once the farms had been identified, the investigator traveled to the farm within 2 to 5 days from onset of clinical signs. The clinical history of the outbreak was reviewed and recorded after discussing it with farm personnel. Attempts were made to collect a complete clinical history of the affected groups as well as assigning a clinical score based on severity of respiratory signs. Scores ranged from 1 to 3 where 1 = cough in less than 25% of the pens, 2 = cough and sneezing in 25 to 75% of the pens and 3 = cough, sneezing and lethargy in 75% or more of the pens. If there was more than one group of pigs affected in a farm, the group with the most recent onset of clinical signs was selected for testing.

### Air Sampling Procedures and Sampling Scheme

Air samples were collected using a liquid cyclonic collector (Midwest Micro-Tek, Brookings, SD, USA) capable of processing 400 L/min of air. This device has been previously validated for the collection of swine respiratory pathogens including PRRSV, *M. hyopneumoniae* [23], [24] and IAV [27]. Briefly, 10 mL of a minimum essential medium (MEM) solution supplemented with 4% bovine albumin serum (BAS) were added to the liquid cyclonic collector collection vessel. The cyclonic collector was run for 30 minutes allowing airborne particles to be mixed with the collection media solution. Once air sampling was completed, a sterile syringe (Tyco-Healthcare, Kendall Monoject, Mansfield, MA, USA) was used to recover and place the sample in a plastic vial (Thermo scientific capitol vial, Fisher Scientific, Waltham, MA, USA). Air samples were then stored on ice until they were transported to the laboratory for diagnostic procedures. The device would then be cleaned and disinfected according to a previously validated protocol by spraying alkyl dimethyl benzyl ammonium chloride (Lysol, Reckitt Benckiser, Wayne, NJ, USA) on the turbine and the collection vessel. These two surfaces were then sprayed with water to remove remaining disinfectant and dried with paper towels (Kim wipes, Kimberly-Clark, Roswell, GA, USA) [27].

Upon arrival at the farm and confirming the presence of clinical respiratory disease, the first set of samples was collected inside the barn. Fifteen, 30 minute air samples were collected by simultaneously placing four or five cyclonic collectors equally distributed throughout the barn. Cyclonic collectors were placed 1.5 m above the floor and 1 m below the ceiling and secured to a feed line using rubber bungee cords. Pigs did not have direct access to the air collection devices. Power extensions were used to supply power to the air sampling devices.

Immediately after collecting inside samples, samples of the exhausted air were collected. Fifteen, 30 minute samples were collected by placing the cyclonic collectors as close as possible to the draft of air exhausted from the pig barn (farms 1 and 2). Cyclonic collectors were placed either on the ground when samples were collected from exhaust manure pit fans or were hung from a tripod when samples were collected from an external wall exhaust fan. Air sampling devices were run simultaneously.

In farms 3 and 4, there were 15 air samples collected inside the barn, 30 air samples at the exhaust location and 60 samples downwind for a total of 105 air samples per farm. Downwind samples were collected after completing collection of exhaust samples and collection started at the location closest to the farm and ending at the farthest location. For the collection of the downwind samples on the following day, sampling was reversed starting at the farthest location, followed by the closest location and ending sampling at the exhaust collection point. Exterior samples were collected for two consecutive days first at dusk and into the night (first day), and at dawn into the morning (second day) to increase the chances of virus detection [23]. Samples were collected between 0.9 and 2.1 Km downwind from the infected pig population. Google Earth Map (Google, Mount View, CA, USA) was used to identify potential sampling locations based on wind direction obtained through www.weather.com. Potential sampling locations were identified along the closest two roads crossed by the downwind. Upon arrival at these locations, a wind vane together with meteorological information from the website mentioned above was used to identify and confirm the direction where the wind was blowing from. The number of sampling locations was determined based on wind direction changes, therefore, between three and four locations were identified for farms 3 and 4. Sampling locations were not static due to changes in wind direction. Five cyclonic collectors were distributed along the side of the road covering a linear distance of 20 m and run simultaneously. The collectors were placed at distances ranging from 1 m to 1.85 m above the ground and connected through cord extensions to a power source located in the study vehicle.

### Environmental Conditions

Temperature (°C), relative humidity (%) and light intensity (watts/m^2^) were recorded every minute using a weather station (HOBO, Onset Computer Corporation, Bourne, MA, USA) while air was being collected at the external locations at farms 3 and 4. The weather station was located between two cyclonic collectors.

### Pig Population IAV Status Confirmation

To confirm that the population exhibiting respiratory clinical signs was undergoing an influenza epizootic 15 oral fluid samples (saliva) were collected. Oral fluids have proven to be a sensitive method to detect IAV at the population level [32]. Oral fluids were collected as described previously [28]–[32] by hanging 0.6 m of cotton rope from the pen division horizontal bars underneath where the cyclonic collectors were hung. Pigs were allowed to chew on the rope for approximately 30 minutes. At the end of sampling, oral fluids were obtained by placing the rope in a plastic bag (Ziploc bag, S.C. Jonhson & Son, Inc. Racine, WI, USA) and squeezing it until fluid would be deposited in the bottom of the bag. A 10 mL aliquot was transferred to a tube (Thermo scientific capitol vial, Fisher Scientific, Waltham, MA, USA) from each bag and refrigerated until it was transported to the laboratory.

### Diagnostic Testing

All air and oral fluid samples were tested at the University of Minnesota Veterinary Diagnostic laboratory for influenza A RNA by a RRT-PCR targeting the matrix gene [33]. Samples that yielded a cycle threshold (ct) value below 35 were considered positive whereas those that yielded a ct value between 35 and 40 or higher than 40 were considered suspect or negative respectively. Samples that were RRT-PCR positive, were further tested using virus subtyping, quantitative RRT-PCR, virus isolation using MDCK cells and sequencing [30], [34].

A quantitative PCR was developed to quantify the amount of virus particles present in the air samples. Briefly, a partial matrix 1 gene from A/swine/Minnesota/07002083/2007 (H1N1) [GenBank FJ611901, nt 1-387] was synthesized and cloned into pIDTBlue plasmid vector (Integrated DNA Technologies). The plasmid was linearized with SmaI (New England BioLabs), purified with MinElute Reaction Clean Up Kit (Qiagen) and transcribed into RNA with the T7 RiboMAX Express Large Scale RNA Production System (Promega) according to the manufacturer’s instructions. Template DNA was degraded with 5 U RNase-free DNase (Promega) and RNA transcripts were purified once with RNeasy Mini Kit (Qiagen). RNA was quantified by spectrophotometer, aliquoted and frozen at −80°C. RT-PCR was carried out on RNA transcripts with SuperScript III One-Step RT-PCR with Platinum Taq High Fidelity (Invitrogen) containing 5 pmol of each of the following primers pIDT_Matrix_F 5′- CCTAAGATGAGTCTTCTAACCGAGG -3′ and pIDT_Matrix_R 5′- GGGGCCCATGCAACTGG -3′, 1 µl RNA, 9.5 µl nuclease-free water, 12.5 µl 2×reaction mix and 1 µl SuperScript III RT/Platinum Taq High Fidelity Enzyme mix. The reaction was carried out at 45°C for 30 min, followed by 94°C for 2 min, then subsequent 30 cycles at 94°C for 15 sec, 53°C for 30 sec, 68°C for 1 min with final extension at 68°C for 5 min. RNA sequence integrity was checked by sequencing RT-PCR product. Tenfold serial dilutions of transcript RNA (1.34×10^10^–1.34×10^−2^ copies/µl) were used for the determination of detection limits and amplification efficiency of assay.

RNA was extracted from samples using MagMAX 96 Viral RNA Isolation Kit (Ambion) according to the manufacturer’s instructions. Briefly, 50 µl of each sample was used for the extraction of viral RNA. The RNA was eluted with 50 µl elution buffer and stored at −80°C. Real time RT-PCR was carried out in a 25 µl mixture using the AgPath-ID One-Step RT-PCR reagent kit (Ambion) as listed in National Veterinary Services Laboratories SOP-BPA-9034.03 containing 5 µl RNA, 12.5 µl 2× buffer, 1.0 µl 25× enzyme mix, 1.67 µl detection enhancer, 5 pmol of each primer and 1.5 pmol of probe in a LightCycler 480 (Roche). The reaction was carried out at 45°C for 10 min, followed by 95°C for 10 min, then subsequent 45 cycles at 95°C for 15 sec then 60°C for 45 sec. Fluorescence was recorded at 60°C. CT value and copy number/mL of each sample was calculated by averaging results from 3 replicates.

### Swine Bioassay

To determine whether viral particles contained in the downwind samples were infectious, samples were inoculated into serologically influenza negative pigs. A 2 mL aliquot per pig was used for intra-tracheal inoculation in anesthetized pigs housed at the University of Minnesota animal isolation facilities. Pigs were monitored through nasal swab sampling on days 3, 4, 5 and 6 post-inoculation by RRT-PCR. Pigs were humanely euthanized on day 7 post-inoculation. At necropsy, a tracheo-bronchial swab and lung tissues were collected for RRT-PCR testing. Trachea and lung sections were also examined for histopathological lesions.

### Statistical Analyses

Kruskall Wallis test was used to compare the IAV RNA copies value between oral fluids, air samples collected inside the barn and at the exhaust fan. Repeated measures logistic regression was used to evaluate whether meteorological factors and sampling distance were associated with detection of IAV in outside air samples. Backward stepwise procedures were used for model building including variables that had a P<0.25 in the univariate analysis. Clustering was taken into account in the model for samples collected during the same window of time. Variables that were not normally distributed as assessed by normal probability plots and the Shapiro-Wilk test were log-transformed (distance, temperature, relative humidity, solar radiation). Differences were considered statistically significant when P<0.05. All analyses were performed using SAS 9.2 (SAS Institute Inc., Cary, NC, USA).

## Results

### Detection of IAV by RRT-PCR Inside Barns, at the Exhaust Fans and Downwind

Table 1 summarizes the populations tested according to farm type, age, group size, barn air volume, clinical score, date of sampling and days between onset of clinical signs and investigator’s visit.

**Table 1:** Farm summary and clinical characteristics of the populations tested.

Farm	Farm type	Age of pigs(wks)	Number of pigs per air space	Air volume (m^3^)per air space	Clinical score*	Days between onset of clinical signs and air sampling	Month of year, 2011
1	Nursery	7	1,456	1,082	3	2–4	April
2	Wean-to-finish	13	1,095	1,726	1	7–10	April
3	Wean-to-finish	7	2,198	1,726	2	4–6	September
4	Wean-to-finish	15	1,200	1,987	3	3–5	October

* 1 = cough and sneezing in less than 25% of the pens, 2 = cough and sneezing in 25 to 75% of the pens and 3 = cough and sneezing in more than 75% of the pens.

Table 2 summarizes the IAV RRT-PCR results from oral fluids and from inside and exhaust air samples. All four farms tested IAV positive by RRT-PCR in oral fluids. Three out of four farms yielded positive IAV results in air samples both inside the barn and at the exhaust point. Farms 1, 3 and 4 which were sampled shortly after the acute clinical signs were reported, had the highest number of positive samples and an average viral load of 3.20E+05 RNA copies/m^3^ for inside air samples and 1.79E+04 RNA copies/m^3^ for outside air samples. Ct values in farm 2 were considered suspect and virus levels could not be quantified. A summary of results by farm can be seen in Table 3. H1N1, H1N2 and H3N2 IAV subtypes were detected from both oral fluid and air samples.

**Table 2:** Number of positive and percentage of influenza A virus (IAV) RRT-PCR and virus isolation results from oral fluid samples and air samples collected inside the barn and at the exhaust fan from acutely infected pig populations.

	Oral fluids		Inside air samples		Exhaust air samples		
Farm	RRT-PCR	Virus Isolation	RRT-PCR	Virus Isolation	RRT-PCR	Virus Isolation	IAV subtype
1	15/15* (100)	11/15 (73)	15/15 (100)	6/15 (40)	15/15 (100)	1/15 (7)	H1N2
2	15/15 (100)	NT	0/15 (0)	0/2 (0)	0/15 (0)	0/2 (0)	H1N1
3	12/15 (80)	0/5 (0)	13/15 (87)	0/5 (0)	20/30 (67)	0/5 (0)	H1N1
4	15/15 (100)	5/5 (100)	15/15 (100)	1/5 (20)	26/30 (87)	0/4 (0)	H3N2

* Number of positive/total number of samples (percentage).

NT = not tested.

**Table 3:** Average influenza A virus (IAV) load and standard deviation (SD) values for oral fluids (RNA copies/ml) and air samples (RNA copies/m^3^ of air) collected from four infected pig populations.

	Oral Fluids		Inside Air		Exhaust Air	
Farm	Mean*	SD	Mean	SD	Mean	SD
1	NA**	NA	8.54E+05^a^	2.04E+05	6.35E+04^b^	3.30E+04
2	2.77E+04	2.31E+04	0	0	0	0
3	3.46E+05^a^	3.41E+05	2.20E+04^b,c^	1.35E+04	1.27E+04^c^	9.63E+03
4	5.71E+07^a^	3.74E+07	8.32E+04^b^	4.57E+04	1.01E+04^c^	9.04E+03
Total***	2.75E+07	3.85E+07	3.20E+05	4.01E+05	1.79E+04	2.49E+04

Means within row with different superscripts indicate statistically significant differences (*P*≤0.05).

* Mean quantitative RT-PCR values for positive samples only.

** NA: Non available.

*** Farm 2 excluded from air totals.

Results from downwind air sampling are summarized in Table 4. Samples with a detectable level of IAV genetic material ranged between 1 to 8E+03 RNA copies/m^3^. Virus levels were similar across the distances sampled and there were no differences in the levels of virus detected in samples collected at dawn or dusk (results not shown). Complete subtyping or partial subtyping was obtained for downwind samples as shown in Table 4.

**Table 4:** Number of influenza A virus (IAV) positive samples, IAV farm subtype and average IAV RNA copies/m^3^ of air collected from downwind samples.

Farm	Distance (Km)	<35 Ct	35–45 Ct	>45 Ct	Average RNA copies/m^3^ of air (*)	Subtype
3	1.2	0/14&	1/14	13/14	6.17E+03	H1N?, H?N1
^3^	1.8	0/30	10/30	20/30	4.49E+03	Untypable
^3^	2.1	1/15	3/15	13/15	8.58E+03	Untypable
^4^	0.9	0/14	5/14	9/14	1.74E+03	Untypable
^4^	1.5	2/15	7/15	8/15	3.43E+03	H3N2, H?N2, Untypable
^4^	1.6	2/15	8/15	7/15	4.17E+03	H?N?, H3N?
^4^	1.9	0/14	6/14	8/14	6.83E+03	Untypable

& Number of positive/total samples tested.

Ct: Cycle threshold value.

(*) Average of positive qRT-PCR results only.

? = Untypable.

Downwind air sampling in farms 3 and 4 yielded a total of 5 and 34 positive and suspect RRT-PCR results, respectively. The positive air sample from farm 3 was untypeable, whereas two of the suspects yielded partial subtype (e.g. H1N-untypable and an H-untypable N1) information. On farm 4, two of the four positive samples yielded either a complete (e.g. H3N2) or a partial (e.g. H-untypableN2) subtype (Table 4).

In general, average ct values differed significantly (P<0.05) among oral fluids, inside air and exhaust air samples with ct values being lowest in oral fluids and highest in exhaust air samples (Table 3).

### Virus Isolation and Sequencing

In farm 1, IAV was isolated from oral fluids (n = 11), inside air (n = 6) and exhaust air (n = 1). In farm 4, virus was isolated from oral fluids (n = 5) and inside air (n = 1), but virus was not isolated from exhaust air samples. No viruses were isolated from samples in farms 2 and 3. Sequencing results from oral fluids and air samples was possible only in farms 1 and 4 and these shared ≥99% HA virus sequence similarity within each farm (Table 5). None of the downwind RRT-PCR positive air samples was virus isolation or swine bioassay positive.

**Table 5:** Average meteorological conditions and standard deviation (SD) values for farms 3 and 4 at the time of sample collection.

Farm 3				Farm 4			
Sample	T (Celsius)	RH (%)	Solar Radiation(W/m^2^)*	Sample	T (Celsius)	RH (%)	Solar Radiation(W/m^2^)
Inside	NA	NA	NA	Inside	20 (1.8)	53.3 (4.5)	0 (0)
Exhaust PM	21.1 (0.8)	41.2 (2.7)	186.8 (104.5)	Exhaust PM	9.3 (1.1)	58.6 (3.5)	46.2 (21.4)
PM 1.2 Km	16.1 (0.2)	46.3 (1.5)	0 (0)	PM 1.5 Km	6.8 (0.6)	64.8 (2.3)	0 (0)
PM 2.1 Km	15.2 (0.2)	52.7 (1.8)	0 (0)	PM 1.9 Km	5.6 (0.2)	69.7 (0.7)	0 (0)
AM 1.8 Km	9.5 (2.8)	78.9 (8.3)	259.5 (229.5)	AM 1.6 Km	−1.2 (2.9)	81.9 (7.8)	53.5 (97.1)
Exhaust AM	20.7 (1.4)	44.7 (2.9)	519.4 (322.2)	AM 0.9 Km	7.5 (1.2)	62.5 (3.7)	453.6 (43.9)
				Exhaust AM	2.1 (1.1)	85.5 (1.9)	8.6 (5.3)

NA: Non available.

* Zero values indicate that samples were collected at night.

### Meteorological Conditions and Detection of Influenza in Air Samples

Table 5 summarizes the meteorological conditions for farms 3 and 4 at the time of sampling. No estimates were obtained from the repeated measures logistic regression model due to low power.

## Discussion

Understanding the routes for IAV transmission is vital for designing appropriate IAV control strategies and prevention of zoonotic infections. In particular the role that aerosols play in IAV transmission in pigs and the risk they represent to people has not been fully elucidated. This study provides information on IAV aerosols generated by pigs under field conditions. In this study we detected infectious IAV and quantified the amount of virus present in air samples collected from the interior and at the exit point of swine barns. Furthermore, influenza genetic material could also be detected downwind from the infected population for distances up to 2 Km. Three commonly found subtypes of IAV in pigs were detected in the air samples collected at the various locations and the IAV HA sequences identified in the swine oral fluids matched the sequences in aerosols providing evidence that the viruses detected in the aerosols originated from the pigs in the study. Overall our results indicate that pigs can be a source of IAV infectious aerosols and that these aerosols can be exhausted from pig barns and transported downwind.

IAV isolation from air samples was possible in two of the four farms. Isolations were successful in samples collected inside the barn and at the exhaust point indicating that short distance aerosol transmission is possible for IAV. Our findings support previous reports where exposure to infectious aerosols is considered a significant route of transmission within swine populations [6]. Our results also suggest that swine aerosols can be a source of infectious virus for people. Personnel working with pigs have been shown to be at higher risk of IAV infections of swine origin [40], [41]. Overall our results also indicate that risk of infection is higher when exposure occurs to contaminated aerosols generated within confined enclosures and immediately exhausted from those enclosures but the risk decreases significantly as the aerosols disperse away from the facilities as discussed below.

Detection of IAV genetic material from downwind locations was possible for as far as 2.1 km from the source population and although we were not able to isolate viable IAV, we speculate that regional airborne dissemination of IAV in pigs is possible under the appropriate environmental conditions. Several epidemiological studies had found an association between swine farm density and IAV seropositivity [7], [8] and airborne transmission was suspected in the detection of H1N1 and H3N2 IAV infections in Minnesota turkey premises [9]. Furthermore, dissemination of equine influenza within a 1–2 km region was attributed to airborne spread associated with wind direction, temperature and relative humidity [42], [43]. Our difficulty to isolate the virus from downwind air samples was most likely due to the amount of virus present in the sample as the viral load significantly decreased with distance from the source population. This was reflected in the quantitative PCR results. Furthermore, isolation of infectious pathogens from air samples is in general poor due to the physical disruption of the pathogens [35]–[37]. Alternatively environmental conditions could have inactivated the virus. In addition, enclosed environments offer better conditions for particle saturation due to limited drafts whereas conditions outside facilities favor the mixing with air drafts which dilutes the concentration of viral particles [12], [38], [39]. Given that only 4 farms were tested in our study (only 2 for the downwind testing), we did not have enough power to assess patterns of regional spread or association with environmental conditions.

The populations selected for this study were conveniently selected for presenting an acute outbreak of influenza infection. This was done to increase our chances of airborne virus detection. Virus was not detected in farm 2 which was tested after the acute clinical signs had disappeared. Both, time to onset of clinical disease and presence (or lack of) of immunity are associated with detection of virus in the air [26], [27]. Both, acutely and endemically infected populations are common and the relative role that such populations have in IAV transmission to pigs, people or other species needs to be further elucidated. Transport of IAV in the air might also be associated with co-infections which may increase the likelihood of generating infectious aerosols, but this was not assessed as part of this study and needs further investigation.

To our knowledge this is the first study that has quantified the load of influenza virus in aerosols generated by pigs under field conditions. As expected, we found higher levels of viral genetic material in air inside facilities and these levels decreased in air immediately exhausted from the facilities although they remained high. However, viral levels dropped significantly in downwind samples with most of the samples testing negative indicating that IAV was not uniformly distributed in that air. Overall, viral levels varied among farms, clinical status of the pigs and distance to source population. How the levels of viral genetic material relate to risk of transmission to other pigs or other species including humans needs further research and it is beyond the scope of this study.

In conclusion, this study provides new information into the understanding of IAV aerosol generation in pigs and the role that infectious aerosols play on airborne transmission. Our study is the first to report that pigs acutely infected with IAV release viral particles into barn airspace that can also exit the building and be transported downwind. More importantly, some of these viral particles are infectious posing a risk to other pigs and perhaps to other animal species and people. The distance that IAV can be transported through the air as well as whether viable virus can be isolated from long distance air samples remains to be resolved as it will depend on environmental conditions as well as pathogen load and diagnostic methods. Furthermore, the data from this study also emphasizes the need to generate biosecurity mechanisms by which airborne pathogens are prevented from exiting livestock facilities.
